# Detailed analysis of habitat suitability curves for macroinvertebrates and functional feeding groups

**DOI:** 10.1038/s41598-022-15096-8

**Published:** 2022-06-24

**Authors:** Ewelina Szałkiewicz, Tomasz Kałuża, Mateusz Grygoruk

**Affiliations:** 1 Department of Hydraulic and Sanitary Engineering, Institute of Environmental Engineering, Warsaw University of Life Sciences-SGGW, ul. Nowoursynowska 166, 02-787 Warsaw, Poland; 2 Department of Hydraulic and Sanitary Engineering, Poznan University of Life Sciences, ul. Wojska Polskiego 28, 60-637 Poznań, Poland; 3 Department of Hydrology, Meteorology and Water Management, Institute of Environmental Engineering, Warsaw University of Life Sciences-SGGW, ul. Nowoursynowska 166, 02-787 Warsaw, Poland

**Keywords:** Hydrology, Freshwater ecology, Environmental impact

## Abstract

Environmental flows have primarily a practical purpose, being an important part of water management. Despite the widespread use of environmental flows, current studies rarely describe practical insights of the methods or consider environmental flows based on ecological traits, especially regarding macroinvertebrates. In addition to hydraulic parameters, the ecological traits may also indicate processes that drive the distribution of organisms. Nevertheless, so far the habitat suitability criteria for functional feeding groups, the most commonly used ecological trait for macroinvertebrates, have not been described. In this study, we performed a detailed analysis of habitat suitability curves for the macroinvertebrate community and for FFGs. The criteria were determined based on data collected during two field campaigns (2018 and 2019) from the Flinta River, a lowland, dynamic, sandy stream located in western Poland. The method of habitat preference curves (HPCs) for flow velocities, depths and substrate was adopted. Before determining the final habitat suitability criteria, for all considered groups the habitat preference curves and habitat utilization curves were determined separately for the data collected in 2018 and 2019. The results showed that this step was key in developing the final habitat suitability criteria. Additionally, considering FFGs provided insight into the mechanisms that drove the distribution of organisms and resultant suitability.

## Introduction

The flow regime plays a key role in maintaining and protecting aquatic ecosystems, as described by the natural flow regime paradigm^1^. The overall effect of the natural flow regime on aquatic ecosystems was summarized in four principles described by Bunn and Arthington^2^. First, flow (described as the volume of water moving through the cross-sectional profile of the river channel in a given time) is a major determinant of habitat availability in streams, which in turn influences the composition and distribution of the biota. The magnitude of flow, together with the geological structure of the substrate, interacts with sediment transport processes that determine river geometry, the formation of riffles and pools, or substrate stability. Additionally, the magnitude of flow affects the amount of nutrients and organic matter transported along the channel and between the channel and floodplains. Second, aquatic species have adapted their life history strategies in direct response to the natural flow regime. Third, maintaining the natural longitudinal and lateral continuity of rivers is essential to the population viability of many species of aquatic organisms. The life cycles of many species are associated with their capability of migration along the river and between the channel and floodplains. This continuity can be maintained by the cyclical occurrence of high water levels and the absence of engineered barriers. Finally, invasion/succession of exotic and alien species can be facilitated by altering the flow regime, since flow alterations, water damming (changing the current character from lotic to lentic), or connections between catchments support acclimatization of alien species. Thus, disturbances of natural flow regime, or a change in the parameters that characterize that regime (e.g., flow magnitude, frequency), can alter habitat availability, disrupt life cycles of organisms, and affect the structure and well-being of aquatic ecosystems^3,4^. At the same time, water is crucial to human functioning^5^. Meeting individual needs involves water management for human utilities, and agricultural, industrial, or energy purposes. The most severe anthropogenic impact on the flow regime include: (1) reservoirs and water plants that alter flow magnitude and diversity, (2) barriers that reduce connectivity along the rivers and between the channel and the floodplains, and (3) water abstractions that cause significant changes in flow magnitude within the channel^1,2,6–8^. This, together with intensive urbanization and land cover changes within catchments, has led to overexploitation of water resources, disruption of the natural flow regime and significant degradation of aquatic ecosystems^1,2,9,10^.

Among the tools used to ensure a balance between water management and protection of aquatic ecosystems are environmental flows, which have become a permanent part of the legal basis of water policy worldwide^11–13^. According to the Brisbane Declaration^14^, updated by Arthington et al.^15^, environmental flows describe the quantity, timing and quality of flows and water levels necessary to sustain aquatic ecosystems, which in turn support culture, economy, sustainable livelihoods and human well-being. It has been highlighted that environmental flows should be determined based on the results of detailed research on the interactions between the flow characteristics, environmental conditions, and aquatic organisms present in a particular ecological setup. Habitat suitability modeling (HSM) is in line with these insights and has been recognized as one of the currently most reliable tools for determining environmental flows and assessing the impact of hydrotechnical and river restoration projects on aquatic habitats^13,16^. HSM methods are indirectly related to niche theory^17,18^, which assumes that the distribution of species is determined by the fulfilment of three conditions: (1) local environments allow population growth, (2) interactions with other species allow survival, and (3) the location is available given the species' dispersal capabilities. Thus, all organisms have certain preferences for physical parameters that determine specific habitat types. HSM methods build on these preferences by identifying relationships among parameters that determine particular habitat types (e.g., depths, water velocities, cover, substrate type, temperature) and the distribution of aquatic organisms^19^.

Habitat suitability at a given flow (or hydrological regime) can be determined for single species, whole communities, or life stages^20–25^. Currently, most studies on environmental flows have been conducted for fish, and studies on macroinvertebrates in this context are underrepresented^7,26^. Macroinvertebrates have essential functions and are key to maintaining the integrity of aquatic ecosystems^27,28^. They are an important part of the organic matter cycle^27,29^, and the presence or absence of a single species can dramatically alter ecological processes such as rates of grazing and decomposition. There are numerous food-web linkages in which one species interacts positively or negatively with others or in which the addition or loss of a single species alters the food-web dynamics^30^. The functional feeding groups (FFGs) concept classifies macroinvertebrate species based on the mechanism of food intake and type of food^28,29^.Grazers/scrapers use a variety of mechanisms to gnaw and scrape food such as algae. Shredders feed on coarse particulate organic matter (CPOM), breaking it down into fine particulate organic matter (FPOM) and dissolved organic matter (DO). CPOM reaches rivers from terrestrial areas (e.g., as leaves, litter, or woody debris) or comes from macrophytes growing within the river channel. Gatherers are adapted to consume mainly fine particles deposited on the surface of the sediment or in its deeper layers. They also constitute the most abundant group of macroinvertebrates and are often prey for predatory insects. Filter feeders have evolved a variety of mechanisms to capture FPOM suspended in the water. They occur at sites of intense particle transport. They also delay downstream runoff of organic matter. The last group, predators, feed mainly on animal tissue by swallowing their prey or by piercing and sucking out the body contents. Beside their essential role in organic matter processing, macroinvertebrates constitute food sources for both aquatic and terrestrial vertebrate consumers (e.g., fishes and birds)^28,30^. Their traits are also used in biomonitoring^31–33^; for instance, the taxa Ephemeroptera, Plecoptera and Trichoptera (EPT) indicate good water quality due to their high sensitivity to stress.

The requirements of macroinvertebrates as to availability of flow rate may be different from those of fish. Organisms in this group often have smaller ranges of flow requirements than large fish and greater requirements compared to small fish^34^. Additionally, macroinvertebrates are less mobile, and thus cannot escape unfavorable conditions within a catchment, or even a reach, as effectively as fish. Low flows first affect the availability, diversity, and suitability of microhabitats by altering their depth and water flow velocity. Essentially, reducing water velocity increases fine sediment deposition and reduces food supply^35,36^. This in turn affects the composition of the substrate, its suitability for macroinvertebrates and their ability to take up food. Reduced flow can also result in changes in physicochemical parameters of water by affecting the concentration of dissolved contaminants^35,37^ and water temperature (which may increase as a result of faster warming during summer or decrease as a result of significant groundwater recharge to the watercourse)^35,38^. Conversely, temperature changes will be associated with changes in dissolved oxygen^35,38,39^, to which certain taxonomic groups are highly sensitive^38^. In the initial phase of low flows, an increase in macroinvertebrate density is typically observed due to a decrease in habitat availability. Subsequently, competition, predation and limited food supply cause a decrease in the density of organisms^35,39^. Hence, lower habitat diversity due to lowering the water level very often results in a decrease in biodiversity. Species composition also changes, as the number of taxa that prefer slower water flow velocities increases^35^.

Environmental flow analyses assume that the distribution of aquatic organisms is mainly determined by hydraulic parameters and substrate^24,40–42^. Other factors such as temperature, food availability, and interspecific interactions also influence the distribution, density, and community structure of aquatic organisms, but are rarely considered^43,44^. Animals may indirectly respond to hydraulic conditions through other factors that are directly related to flow. Analyses of the ecological processes determining the distribution of individual organisms can be aided by aggregating taxa with similar biological traits and environmental responses into so-called functional or ecological groups^29,45,46^. The mentioned FFGs could facilitate environmental flow assessment for macroinvertebrates by putting the ecological context into the analyses and identification of the most demanding group in terms of flow. There is a correlation between the distribution of organisms and food availability^34,35,42,47^. Furthermore, food is supplied with flowing water; thus the occurrence of particular groups may be correlated with hydraulic parameters^42,47^. Nevertheless, previous studies on environmental flows were performed for whole communities or individual taxa of macroinvertebrates^26,40,48^, but ecological groups so far have not been considered. The flow needs of individual taxa may not cover the full range of water demands for other species occurring within the riverbed. Also, we hypothesize that the water demands assessed based on the macroinvertebrate assemblage may be affected by the most abundant taxa.

The relationship between the physical parameters of the environment and the distribution of organisms is used to determine the so-called habitat suitability criteria, which define the range of suitability of the parameters of a given habitat for the analyzed group of organisms^49^. HSM methods differ in how these criteria (or relationships) are determined. They can be built using habitat suitability curves, fuzzy logic methods, neural networks, or multiple linear regression^19,24,25,50^. The criteria are then used to assess the area of suitable habitats for different flow scenarios. Currently, habitat suitability curves (HSCs) are one of the most popular methods for determining habitat suitability criteria^9,25,50–53^. However, the scientific robustness of HSCs as the core of ecohydraulic flow assessments or restoration tools still remains an open issue^54^. Also scientific studies to establish HSCs as a robust method for characterizing an aquatic habitat for all aquatic species have never been conducted. Nevertheless, habitat suitability curves are widely used in environmental flows, but one can only find a few analyses of habitat suitability criteria, which could help to interpret data, describe practical insights of the method, or summarize its shortcomings, especially regarding macroinvertebrates. For instance, determination and interpretation of HSCs are easier if only the data from a single field campaign are considered^49^. However, doing so may not be possible, due to either technical constraints or the need to consider broader environmental conditions. Subsequently, data from several field campaigns may be aggregated in three ways: (1) combining samples from different watercourses regardless of the sampling season and without data preprocessing, (2) combining samples from different rivers but only for some specific period, usually low flows, and (3) combining samples from different watercourses regardless of the sampling season, after appropriate data processing^55^. From a practical point of view, data collected in different flow conditions may provide crucial information about macroinvertebrate behavior, which can be blurred as a result of data pooling. Another example is to determine the suitability of HSCs. The suitability is usually expressed by a suitability index (SI), which is calculated based on organisms’ abundance in specific habitat parameter values, and ranges from 0 (low suitability) to 1 (high suitability). However, more recently, regarding macroinvertebrates, Theodoropoulos with co-authors applied the K parameter approach, which, beside abundance data, considers indices used to assess the quality of macroinvertebrate community structure^24,26,55^. So far only a limited number of studies have compared the two methods, and it is not known what differences can be found using these methods and what the range of applicability of the K parameter is.

In this study, we aimed to perform a detailed analysis of habitat suitability curves for the macroinvertebrate community and for FFGs, since previous studies for macroinvertebrates were performed mainly for a community or individual taxa. Incorporating FFGs facilitated data interpretation and identification of the most vulnerable group in terms of flow rate. This information may be crucial for the protection of aquatic ecosystems and water management, as choosing the target group of organisms appropriately provides a more robust environmental flow assessment. We also compared the suitability determined using the SI index and the K parameter described by Theodoropoulos et al.^24,26,55^. Although considering the indices that indicate the quality of organisms’ community structure seems to improve the adequacy of habitat suitability curves, similar results at the macroinvertebrate assemblage level were obtained. We concluded that the K parameter may be not appropriate for smaller ecological groups. Finally, to fill the gap in detailed and practical analyses of habitat suitability curves, we analyzed issues related to combining data and practical interpretation of the resultant habitat suitability curves. Our conclusions and insights from analyses for data from two field campaigns may be an important resource for future studies and practical environmental flow assessment in terms of determining final habitat suitability curves.

## Methods

### Study area

**Figure 1:**
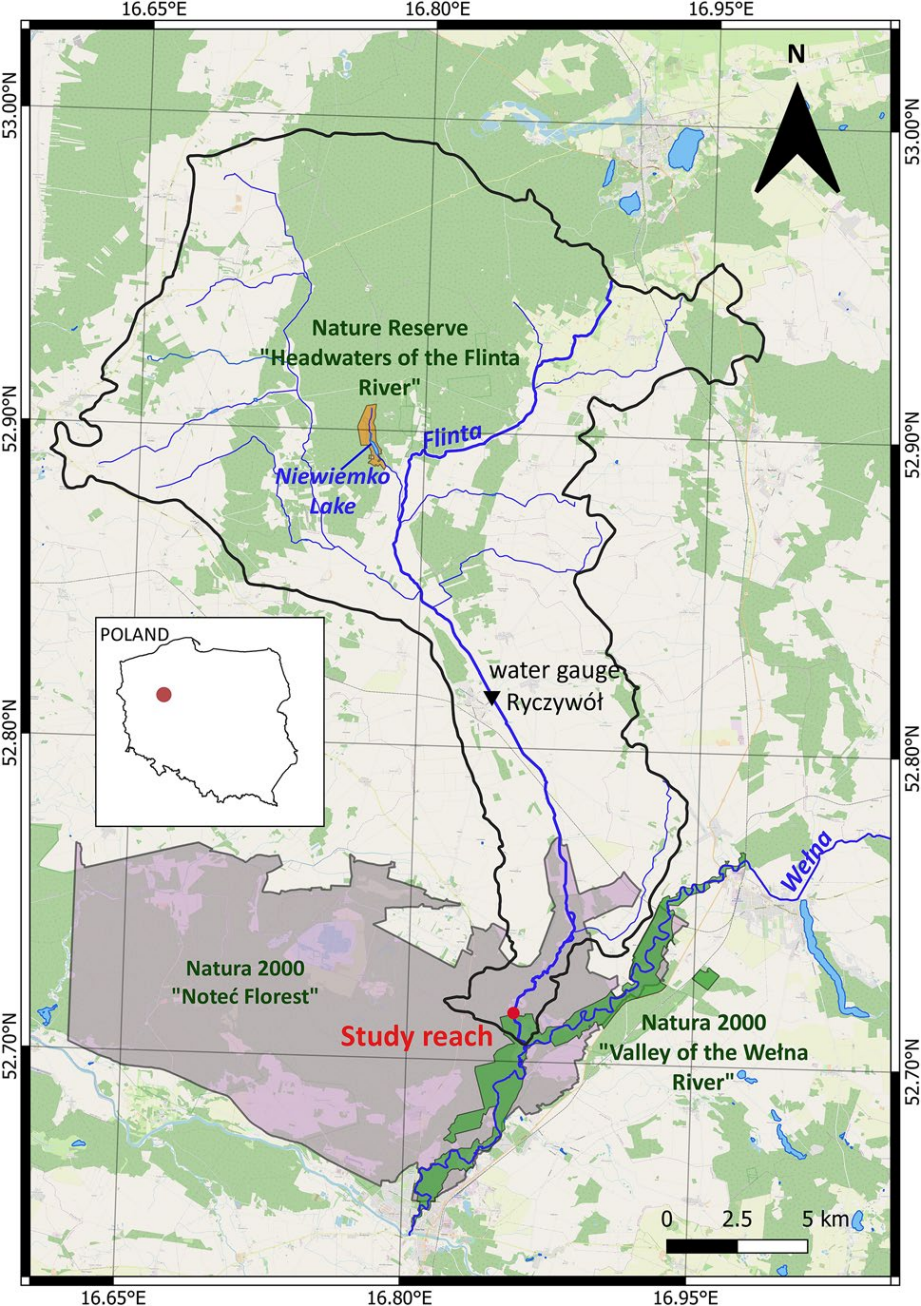
Location of the Flinta River catchment and the study river reach. This original map was created using QGIS 3.20.2 (https://download.qgis.org/downloads/). The layers of rivers, lakes and the Flinta River catchment were obtained from the State Water Holding Polish Waters (https://dane.gov.pl/pl/search?q=MPHP), layers of protected areas were obtained from the Regional Directorate for Environmental Protection in Warsaw (http://warszawa.rdos.gov.pl/dane-i-metadane), and the base map was the OpenStreetMap (https://www.openstreetmap.org/#map=7/52.012/16.414).

The present study was based on data collected from a section of the Flinta River, which is a small, sandy lowland stream located in western Poland (Fig. 1). The total length of the Flinta River is approximately 37.8 km and the catchment area is 338.5 km^2^. The climate is temperate to continental with high variability in weather conditions and four main seasons. Summer is warm with temperatures around 20–25 °C, and winter is cold with an average January temperature of − 4 °C. Average annual air temperature is 7 °C. The highest rainfall occurs in summer and the lowest in winter. The area is characterized by the lowest total annual precipitation in Poland, at about 510 mm. The duration of snow cover varies from 50 to 80 days, and the growing season duration is 220 days^56^. The Flinta River valley is composed of sand^57^. The largest share of land cover within the Flinta River catchment is covered by arable lands (45%), forest (44%) and grassland (10%). The average slope of the riverbed is about 0.75‰, with lower gradients in the upper and middle parts, where the river valley is wide and flat^56^. Along the downstream part of the river, in the length of 11 km, the valley becomes narrower, and the channel slope increases. The river is characterized by a snow-rainfall streamflow regime and inflow of cold waters from the Niewiemko Lake, located in the nature reserve “Headwaters of the Flinta River". The Flinta River is monitored by one water gauge station located at Ryczywół (Fig. 1). Hydrological observation over years indicated that during cold winters, ice phenomena such as frazil ice, border ice and ice cover occur within the river channel of the Flinta.

The selection of the study object was guided by information on technical maintenance works and the condition of hydromorphological elements. The Flinta riverbed was modified in the 19th and early twentieth centuries^58^. For this reason, along the river one can distinguish sections with varying degrees of hydromorphological alterations, from strongly regulated to semi-natural ones, which make the Flinta River an interesting case study for ecohydraulic analyses. The low section of the river is characterized by very good hydromorphological conditions, although at the end of the nineteenth century it was straightened. The cessation of maintenance works, the establishment of protected areas and the relatively high riverbed slope ensured the dynamics of hydromorphological processes and contributed to the self-restoration. Thus, the study section was located about 1.4 km upstream of the river’s confluence to the Wełna River, in the Natura 2000 areas “Noteć Forest” and “Valley of the Wełna River” (from 52.715906 N, 16.856113 E to 52.713913°N, 16.856095 E). It had a length of 300 m, an average width of about 4.5 m, and a slope of 0.99‰. Assessment of hydromorphological elements, performed using the River Habitat Survey method, classified the hydromorphological elements in the highest class of hydromorphological status (class I)^59^. In the studied Sect. 5 types of mesohabitats were identified: riffles, pools, runs, planes and shelves^60^. The dominant substrate was sand; however, gravel was also present within the riffles; and fine sand and silt (characterized by a high proportion of silt and clay fractions and organic matter) were present in the shelves and pools. Within the riverbed wood elements were identified in the form of branches, tree trunks and roots. During the growing season, the riverbed was overgrown with submerged, emerged and floating macrophytes. Alders growing on the riverbanks partially shaded the riverbed. Riparian buffer zones were predominantly overgrown with bushes and herbs. Distant parts of the valley were covered with pine forest. Thus, the environmental conditions along and within the studied river reach provided a variety of microhabitats for macroinvertebrates.

### Data acquisition

Benthic macroinvertebrates used for determining habitat suitability curves were collected during two field campaigns carried out in spring in 2018 and 2019. The total number of macroinvertebrate samples was 20 in 2018 and 30 in 2019. The river discharge during field measurements in 2018 was 0.36 m^3^/s (medium range of flows for the Flinta River) and in 2019 it was 0.11 m^3^/s (range of low water flows). Macroinvertebrate samples were collected from microhabitats using a hydrobiological mesh and then preserved in 95% ethanol. A stratified random sampling design was used with the primary strata being mesohabitat types identified within the study river reach^48,49^, including runs, riffles, pools, planes and shelves. Within each mesohabitat, sampling was randomized within microhabitats of different water depth and velocity range (determined by eye). Sampling effort was roughly proportional to the area of the strata. Physical parameters, depths and water flow velocities in the sampling points of macroinvertebrates were measured using a standard hydrometric stick and a current meter. Vertically averaged water flow velocities were measured at depths of 0.4D when D ≤ 0.75 m or at depths of 0.2D and 0.8D when D > 0.75 m, with an accuracy of ± 0.5% of the read value^26,34,61^. A Valeport model 801 hydrometric current meter was used for the measurements, from which averaged velocities from 10 instantaneous measurements were read. A scaled rod of the hydrometric current meter was used to read the depths at the sampling points, allowing for a measurement accuracy of 1 cm. The substrate was qualitatively determined by visual assessment of the size of mineral fractions. The following categories of substrate were assigned: fine sand, medium sand, fine sand with gravel, medium sand with gravel, gravel, silt. The substrate categories were selected based on grain size in mesohabitat types and transitions between them. For instance, within the riffles gravel and gravel with medium sand dominated, while in the zone between riffles and runs fine sand with gravel was observed. Within the runs medium sand was observed in the thalweg zone and the shores were dominated by fine sand. Silt with fine sand was observed in pools and shelves.

Macroinvertebrates were identified to the lowest possible taxonomic level and counted. In most cases it was the genus or species level. Only individuals from two taxa of the order Diptera were identified to the family level (Simuliidae and Chironomidae). For macroinvertebrates, the family level has been shown to be highly congruent with finer taxonomic resolution^62^. Hence, the accuracy of organism identification for the purposes of this study was considered sufficient. Taxa were assigned to one of the following FFGs based on the AQEM/STAR database: grazers/scrapers, shredders, gatherers/collectors, filter feeders, predators, parasites and others^38,63^. Most macroinvertebrates use several mechanisms for food intake. Hence, the AQEM/STAR database uses a 10-point scale that takes into account the gradient of use of a given mechanism by individual taxa. The total number of points assigned to each FFG type used by an individual taxon is always equal to 10.

### Development of habitat suitability criteria

Due to the fact that data were collected during two seasons, before the development of habitat preference curves, the differences in the structure of macroinvertebrate communities was investigated using the ANOSIM test^38,64,65^. The test compares the mean dissimilarities between groups with the mean dissimilarities within groups. As a result, the R parameter is obtained, with values closer to one indicating dissimilarity between groups, values closer to zero suggesting an even distribution of ranks within and between groups, and values below zero indicating greater dissimilarity within groups than between groups^66^. The dissimilarity matrix was determined using the Bray–Curtis dissimilarity index and the number of permutations was 999. Before calculations, the density of organisms in each sample was reduced to a normal distribution by the ln(1 + density) transformation^20,38^. Analyses were performed in the R program using the ‘vegan’ package^67,68^.

Habitat suitability curves were determined using the methodology described by Jowett et al. (2008) and Bovee (1986) for water flow velocities, depths, and substrate categories^52,69^. Category III curves, i.e. habitat preference curves (HPCs), were applied. HPCs were determined based on habitat utilization curves (HUCs) and habitat availability curves (HACs). HUCs reflect the use of habitats by organisms at the time the measurements were taken. HACs determine the amount of available habitats within the analyzed river reach at the time of measurements. By including both components, habitat preference curves determine the probability with which a habitat will be selected if offered on an equal basis with others^70^. Organism preference, which is the ratio between habitat use and habitat availability, was calculated from the following formula^52^:

1 $$P_{r} = \frac{{P\left[ {E/F} \right]}}{P\left[ E \right]}$$

where P_r_ is the relative preference index of organisms for a specific set of environmental conditions (reflects the values of the habitat preferences curve), P[E/F] is the probability of occurrence of specific environmental conditions as reflected by the abundance of organisms (values of the HUCs), and P[E] is the probability of occurrence of specific environmental conditions in the analyzed river reach at the time of field measurements (values of the HACs). P[E/F] and P[E] values were calculated on the basis of the following formulas^52,69,70^:

2 $$P\left[ {E/F} \right] = \frac{{u_{i} }}{{\sum u_{i} }}$$

3 $$P\left[ E \right] = \frac{{a_{i} }}{{\sum a_{i} }}$$

where u_i_ is the total abundance of organisms in the habitat parameter interval i (e.g., abundance of organisms in the water flow velocity interval 0.2 m/s to 0.25 m/s); Σu_i_ is the total abundance of organisms in all intervals; a_i_ is the area of habitats in the habitat parameter interval i; Σa_i_ is the total area of habitats sampled. The habitat suitability index values (SI), ranging from 0 to 1 for HUCs and HPCs, for each habitat parameter were obtained by dividing the P_r_ index by its maximum value^69^.

Due to fact that data from different measurement campaigns were included, prior to calculations the abundance of organisms in each microhabitat was standardized by dividing it by the highest abundance recorded in a given measurement campaign. Subsequently, yet before combining the data, we analyzed the HPCs and HUCs separately for the data collected in 2018 and 2019. One of the assumptions associated with the habitat selection function is that organisms have free and equal access to all available resources^70^. This assumption may be violated if data acquired during low flows are combined with data acquiredduring higher flow values (the same conditions are not available during both periods). Therefore, in such cases, it is suggested to first analyze the data separately for both periods^49^. Then, the final habitat suitability curves, after pooling the data, were determined considering the information obtained from HPCs and HUCs separately for 2018 and 2019^52,69^.

The values of the HSCs were also compared with the values of the K parameter described by Theodoropoulos and co-authors^24,55^. The K parameter, similarly to the SI index, determines habitat suitability in terms of the environmental parameters on a scale from 0 to 1. However, in addition to abundance, it also takes into account indices used to assess the quality and state of macroinvertebrate community structure. The normalized K value for each microhabitat was calculated from the following equation^55^:

4 $$K_{i} = \frac{{0,4n_{i} + 0,3H_{i} + 0,2EPT_{i} + 0,1\alpha_{i} }}{{K_{max} }}$$

where K_i_ is the suitability coefficient for the i-th microhabitat for the analyzed parameter; n_i_ is the number of macroinvertebrate families found in the i-th microhabitat; H_i_ is the Shannon–Wiener biodiversity coefficient for the i-th microhabitat; EPT_i_ is the number of Ephemeroptera, Plecoptera and Trichoptera organisms found in the i-th microhabitat; α_i_ is the macroinvertebrate density in the i-th microhabitat; K_max_ is the maximum K value observed across the dataset. The Shannon–Wiener biodiversity coefficient H_i_ was calculated from the following equation^71^:

5 $$H_{i} = - \mathop \sum \limits_{i = 1}^{S} p_{i} ln(p_{i} )$$

where p_i_ is the ratio of the number of individuals of a given taxon to the number of all individuals in the i-th microhabitat; S is the number of taxa.

Habitat suitability criteria were determined for the macroinvertebrate community as well as FFGs, except for parasites (only two taxa identified) and other (taxa in this group may use different types of feeding mechanisms). Taxa were classified into an FFG based on a value of at least 3 on a 10-point scale of gradient of utilization of a given feeding mechanism^63^. A simple categorization consisting of values of 1 (taxon is assigned to FFG) and 0 (taxon is not assigned to FFG) was used.

## Results

### Macroinvertebrate identification

**Figure 2:**
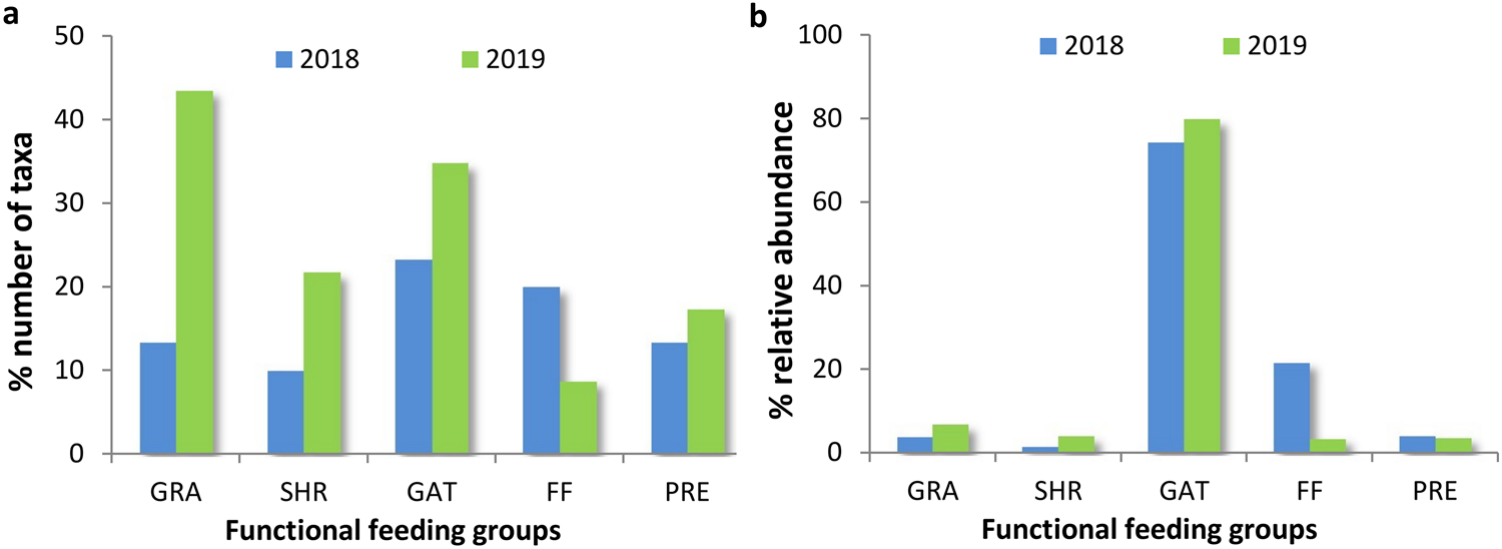
Percentage number of taxa (**a**) and relative abundance of organisms (**b**) in the assigned FFGs. GRA – grazers/scrapers, SHR—shredders, GAT—gatherers, FF—filter feeders, and PRE—predators.

A total of 1217 individuals belonging to 15 orders and 25 families were identified during the two field campaigns (905 individuals in the 2018 samples and 312 in the 2019 samples). The most abundant taxa were organisms belonging to the families Chironomidae and Simuliidae. A total of 107 EPT individuals were recorded (80 in the 2018 samples and 27 in the 2019 samples). The most abundant EPT taxa were *Caenis* sp., *Ephemerella* sp. and *Limnephilus* sp. Considering the percentage of taxa, the dominant FFGs were grazers and gatherers; however, the FFG composition was different in the years sampled (Fig. 2a). Additionally, the relative abundance of organisms showed that the most abundant group was that of gatherers and the number of filter feeders changed significantly between the two seasons (Fig. 2b). The results of ANOSIM analysis showed that the differences in organism structure between samples collected in 2018 and 2019 were statistically significant (*p* = 0.002). However, the R value was low and equaled 0.14 (very small differences between samples). Therefore, it was assumed that the data collected during the two field campaigns can be combined.

### Habitat suitability criteria for macroinvertebrate community

**Figure 3:**
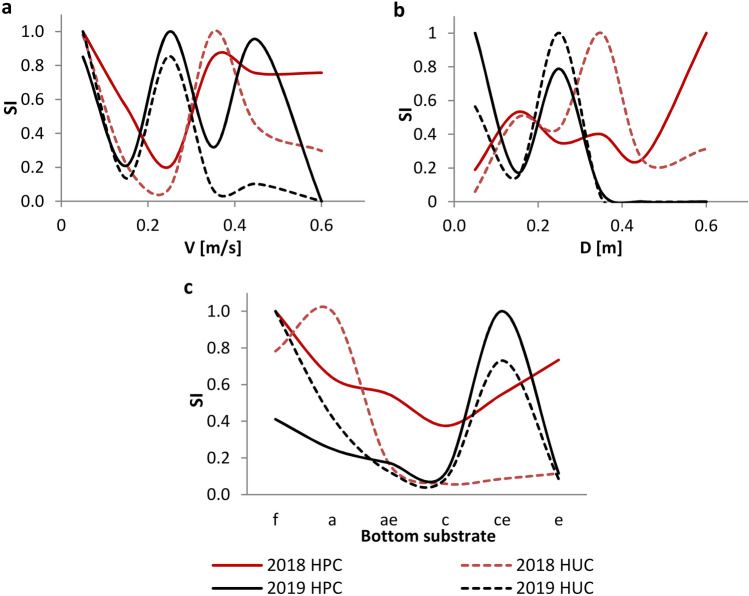
Habitat preference curves (HPCs) and habitat utilization curves (HUCs) for water flow velocities (**a**), depths (**b**), and substrate categories (**c**) separately for samples collected in 2018 and 2019. Categories of substrate: a—fine sand, c—medium sand, ae—fine sand with gravel, ce—medium sand with gravel, e—gravel, f—silt.

The analysis of the HPCs and HUCs determined separately for the data collected in 2018 and 2019 showed that macroinvertebrates tolerate values of velocities ranging from 0.02 to 0.60 m/s and depths ranging from about 0.03 to 0.60 m (Fig. 3). This was evidenced by the shift along the x axis occurring between the curves for 2018 (medium flow value) and 2019 (low flow value). Due to the offset of the HPCs and HUCs for both years, it may be concluded that the range of suitability of flow velocity and depth values was wider than would be apparent from the individual curves. A similar offset would likely be observed if samples were collected during intermediate flows. However, the offset did not occur for the curves for the substrate, which showed that in both years the distribution of macroinvertebrates was similar and ranged from the finest to the coarsest grain sizes.

**Figure 4:**
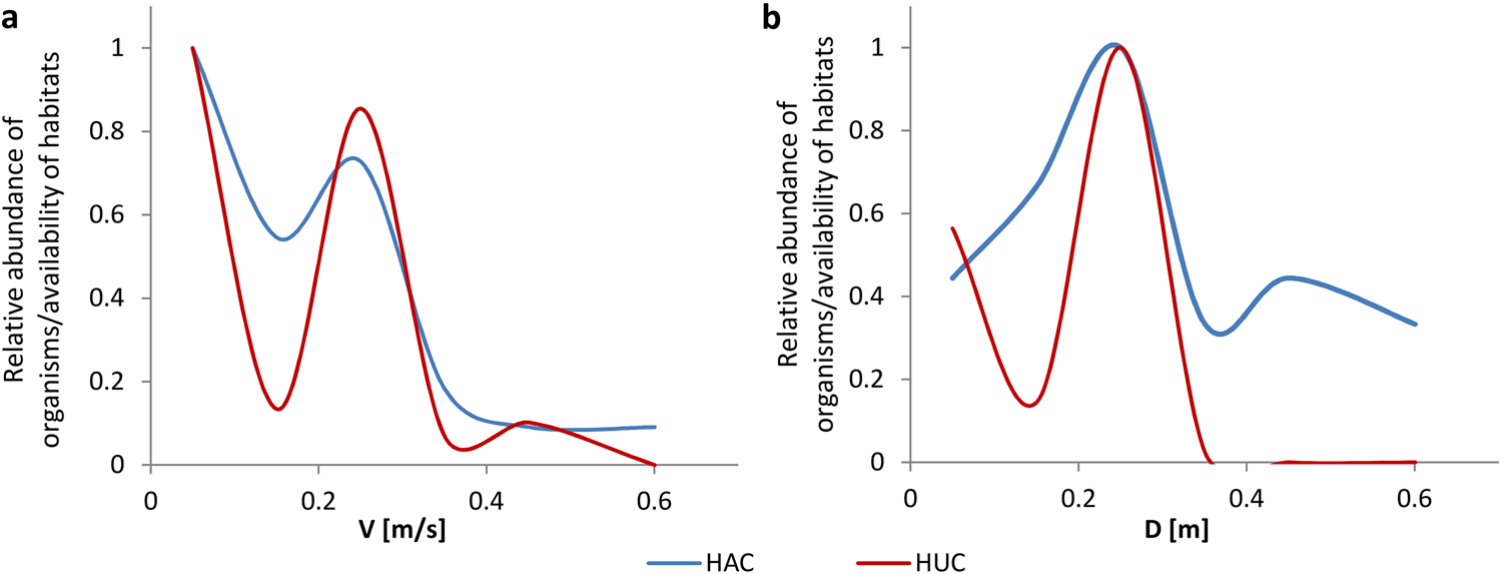
Habitat availability curves (HACs) and habitat utilization curves (HUCs) for the 2019 samples for water flow velocities (**a**) and depths (**b**).

The curves determined for the 2018 and 2019 samples had a bimodal shape (Fig. 3). Bimodality, as stated by Shearer and co-authors (2015), may stem from either the sampling method adopted or the failure to capture all types of available habitats^48^. However, comparisons between the habitat availability curves and habitat utilization curves showed that this is the result of organisms' preference for specific habitat types (Fig. 4). At the inflection points the values of the HACs exceeded the values of the HUCs, meaning that habitats with given values of environmental parameters were sampled, but the abundance of organisms recorded in that habitats was low. Comparing the values of HUCs and HPCs (Fig. 3), the local excesses of the habitat preference curves were revealed. This problem was pointed out by Jowett et al. (2008), indicating that final habitat suitability criteria should be determined based on both curves^69^.

**Figure 5:**
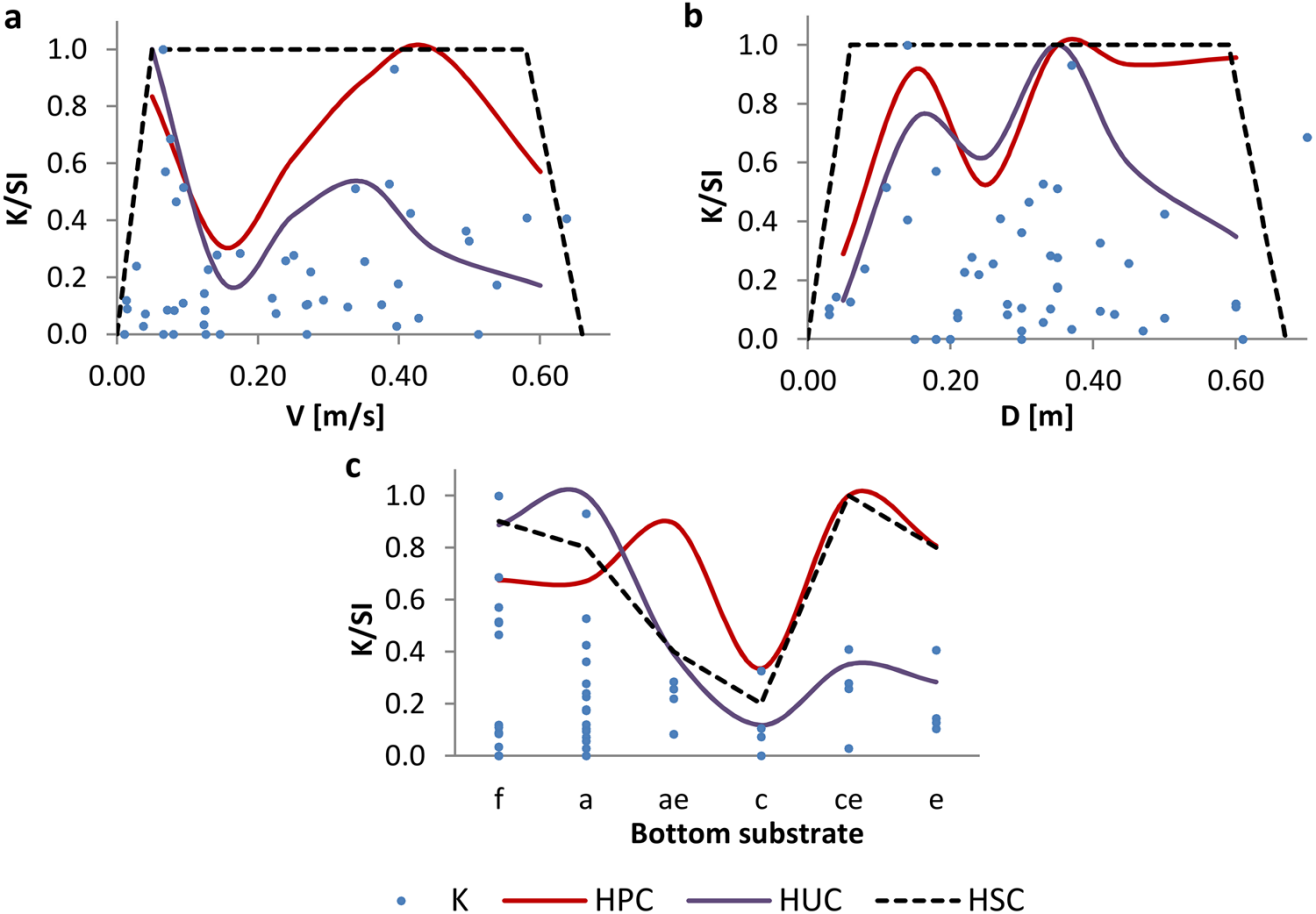
Habitat preference curves (HPCs), habitat utilization curves (HUCs), K parameter values, and final habitat suitability curves (HSCs) for macroinvertebrate community for water flow velocities (**a**), depths (**b**), and substrate categories (**c**). Categories of substrate: a—fine sand, c—medium sand, ae—fine sand with gravel, ce—medium sand with gravel, e—gravel, f—silt.

The values of final habitat suitability criteria (based on combined samples) for the analyzed parameters were determined using the SI index (on HPCs, HUCs and HSCs) and the K parameter (Fig. 5). The results indicated that, despite the differences in the calculation method, the HUCs coincided with the upper limit of the K parameter values, which indicated that similar results were obtained from both methods. For HPCs the discrepancies were larger; however, the K parameter does not take into account information about the number of habitats of a given type available during sampling.

The HUCs and HPCs derived from the combined data, like the curves derived separately for 2018 and 2019, showed a bimodal shape for water flow velocity and depth (Fig. 5). The HPCs showed that the most preferred were values of velocities ranging from 0.05 to 0.10 m/s and from 0.35 to 0.60 m/s, and depths ranging from about 0.15 to 0.25 m and from 0.35 to 0.60 m. However, given the results of the curves determined separately for 2018 and 2019, the highest suitability on the HSCs was assigned for values of velocities ranging from 0.05 to 0.60 m/s and depths ranging from 0.07 to 0.60 m. For the substrate, the values of the HSCs were determined based on the HPCs and HUCs for the combined data. The highest SI values were assigned to the finest material and the material with the largest grain size.

### Habitat suitability criteria for FFGs

**Figure 6:**
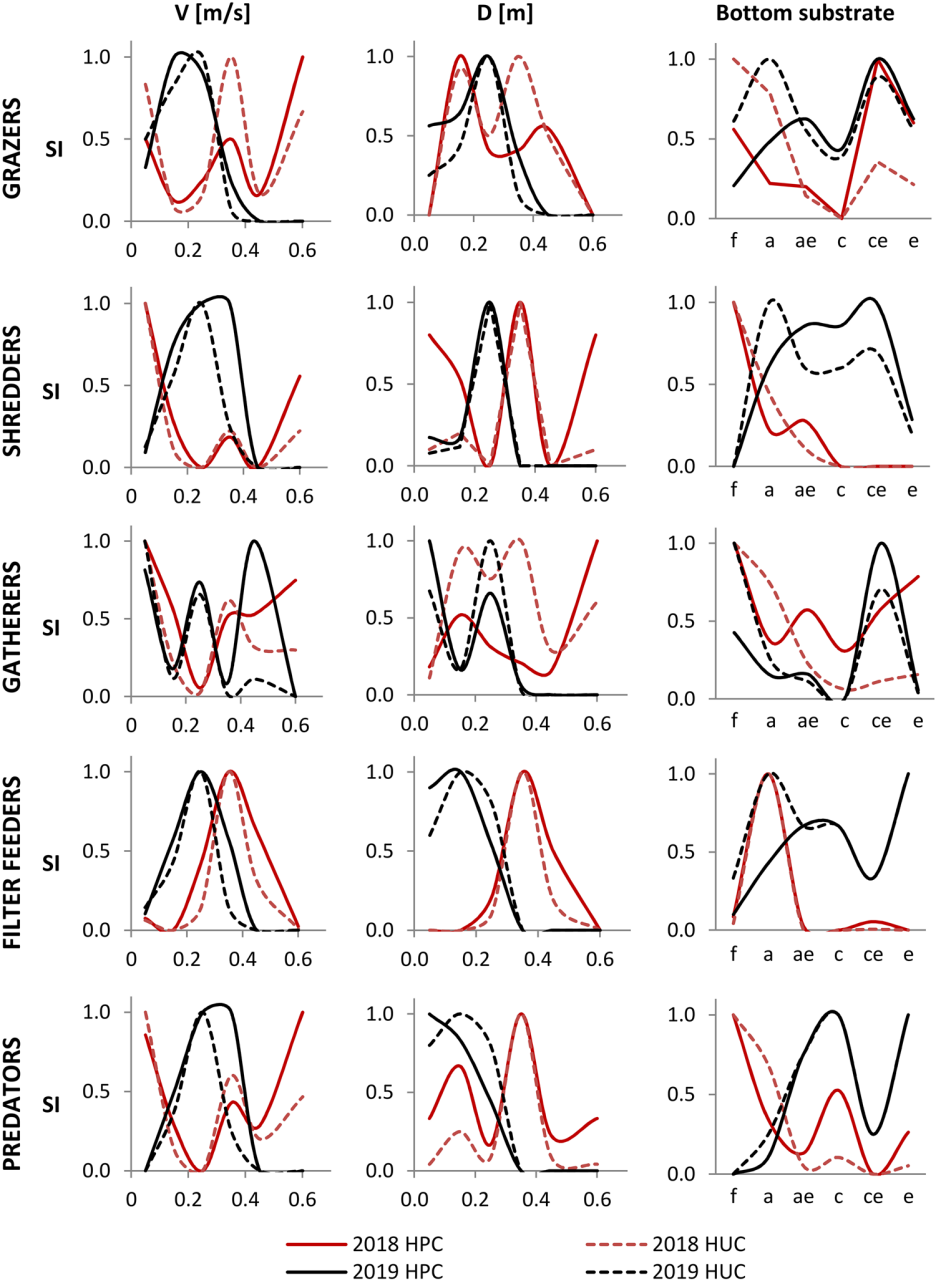
Habitat preference curves (HPCs) and habitat utilization curves (HUCs) for FFGs separately for 2018 and 2019 samples. Categories of substrate: a—fine sand, c—medium sand, ae—fine sand with gravel, ce—medium sand with gravel, e—gravel, f—silt.

The analysis of the HPCs and HUCs determined separately for the data collected in 2018 and 2019 for FFGs showed that, similarly to the data for all organisms, taxa belonging to each group collectively had a wider range of tolerance for water flow velocities and depths than would be apparent from the single curve (Fig. 6). However, the bimodal shape was no longer as pronounced, particularly for filter feeders and the curves for the 2019 data for grazers, shredders, and predators. The narrowest suitability for water flow velocities and depths occurred for filter feeders. The SI index reached the highest values in the values of velocities ranging from 0.20 to 0.40 m/s and depths ranging from 0.15 to 0.40 m. In terms of the suitability of the substrate category, the greatest similarity to the total organisms occurred for gatherers and grazers (the highest suitability of the finest material and the largest grain size). The distribution of the other FFGs was not as closely related to each category of the substrate and changed across the years analyzed. In addition, the curves for the 2019 data for shredders and predators, compared to the curves for 2018, indicated a clear change in suitability regarding flow velocity and substrate (from low and high values of velocities to medium; from fine-grained sediment toward coarser-grained material). It was also noted that the shape of the HUCs and HPCs for predators was similar to the curves obtained for shredders and filter feeders.

**Figure 7:**
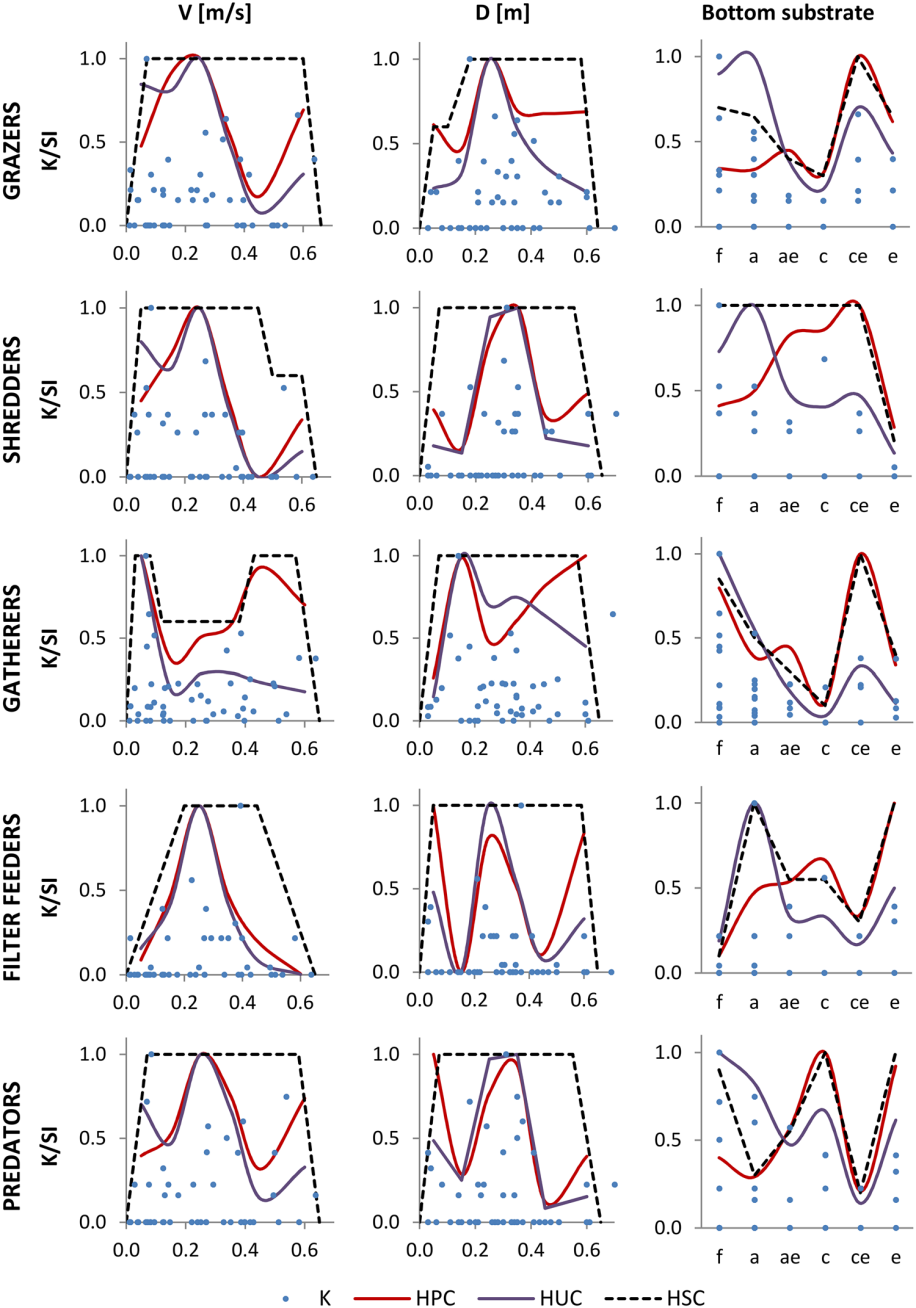
Habitat preference curves (HPCs), habitat utilization curves (HUCs), K parameter values, and final habitat suitability curves (HSCs) for FFGs. Categories of substrate: a—fine sand, c—medium sand, ae—fine sand with gravel, ce—medium sand with gravel, e—gravel, f—silt.

The upper limit of the K parameter and the HUCs for FFGs for combined samples also did not overlap for the macroinvertebrate community (Fig. 7). Perhaps for more specific ecological groups, the biodiversity indices considered for calculating the K parameter are not the best measure for estimating habitat suitability (e.g., the EPT index had a weight of 0.4, and the occurrence of EPT taxa within FFGs was uneven). It is also possible that greater fits would have been obtained with more data. Nevertheless, the final HSCs were determined based on HUCs and HPCs for combined data and the curves determined separately for the data collected in 2018 and 2019.

Similarly to the habitat suitability criteria for the macroinvertebrate community, due to the offset between HUCs and HPCs separately for data from 2018 and 2019, the range of the highest HSC values for flow velocities and depths was extended (Fig. 6). The largest differences in the range of SI index values for the HSC for water flow velocities, compared to the criteria for the macroinvertebrate community, occurred for shredders (SI = 1 for the values of velocities ranging from 0.06 to 0.50 m/s, and SI = 0.4 for the values of velocities ranging from 0.50 to 0.60 m/s), gatherers (SI = 1 for the values of velocities ranging from 0.03 to 0.08 m/s and from 0.43 to 0.57 m/s; and SI = 0.6 for the values of velocities ranging from 0.12 to 0.38 m/s), and filter feeders (SI = 1 for the values of velocities ranging from 0.20 to 0.45 m/s). For depths, habitat suitability criteria were similar to those obtained for the macroinvertebrate community. Interestingly, greater differences were noted for suitability of substrate categories. The HSCs closest to the criteria assigned for the macroinvertebrate community were determined for grazers and gatherers. The greatest range of suitability in relation to the substrate was found for shredders.

## Discussion

Habitat suitability criteria were determined for water flow velocities, depths, and substrate types. Although other environmental variables within the river channel (such as water quality, hyporheic water exchange) also tend to play significant roles in determining habitat quality for macroinvertebrates^32,72^, these three parameters are considered to be good predictors of macroinvertebrate distribution^34,46^. The analysis of the HPCs and HUCs determined separately for the data collected in 2018 and 2019 proved to be an important step in determining the final HSCs. It indicated that the range of tolerances regarding flow velocities and water depths were wider than the curves for combined samples. Considering the data at two different flows and analyzing them separately also minimized the bias that could have resulted from determining SI values on the HSCs at the points where the HPCs exceeded the HUCs. Nevertheless, based on the bimodal shape of the HPCs and HUCs, it could be inferred that organisms mostly preferred three habitat types. The first one presented shallow depths, fine-grained substrate and low water flow velocities. The second one presented shallow depths, coarse-grained substrate, and high water flow velocities. The third one presented large water depths, fine-grained substrate and low water flow velocities. This may be due to the sediment transport processes, substrate stability, and availability of food and refuges^28,34,73^. Lower values of water flow velocity result in deposition of organic matter^74^. Sites with shallower depths are often overgrown with macrophytes, which can provide an additional source of organic matter, substrate for periphyton growth, and shelter from predators^75,76^. Habitats with higher flow velocities are well oxygenated, and coarse-grained material is more resistant to scour in comparison to fine substrate^34^. Additionally, high water flow velocity values provide more food for filter feeders and thus predators^28,29^. The slopes in the HUCs and HPCs could account for the central part of the riverbed or the thalweg zone. One of the assumptions of univariate curves is that the variables (i.e., water depth, flow velocity and substrate type) are independent^52,77^. However, the above-mentioned preferences for habitat types undermine this assumption due to the fact that the variables collectively create specific habitat types. A similar conclusion was reached by Hudson et al.^78^. Additionally, it should be mentioned that bimodality was originally viewed as an error related to a failure in capturing all types of available habitat during data collection^48^, or data stratification, i.e., the division of studied organisms into groups that reflect spatial or temporal changes in microhabitat use patterns (e.g., organisms occurring within riffles or pools)^52^. The former was excluded by comparing HACs and HUCs, but in our study we omitted data stratification. HSCs are typically determined for selected species^40,46,79^, while an entire suite of organisms occurs within the riverbed^54^. Thus, similarly to Theodoropoulos and co-authors^24,26,55^, the community of identified macroinvertebrates was analyzed, and additional groups were separated based on a biological trait, i.e., the feeding mechanism.

Based on the analysis of the HPCs and HUCs determined separately for the data collected in 2018 and 2019, the highest values of the suitability index on the HSCs for the macroinvertebrate community were assigned for values of velocities ranging from 0.05 to 0.60 m/s and depths ranging from 0.07 to 0.60 m. These ranges largely overlap with results from other macroinvertebrate studies. The results of other researchers are summarized in Table 1, indicating those values of water flow velocity and depth for which the highest densities of organisms and suitability were observed. Considering the substrate type, the highest SI values were assigned to fine-grained material (fine sand, silt) and coarse-grained material (medium sand with gravel, gravel). This also coincides with observations and findings reported in the literature^24,34,80^. The comparison of HSCs and literature data indicated that the determination of universal criteria for the same type of rivers can give similar values of the suitability of the analyzed environmental parameters. However, it would require undertaking a meta-analysis of published information in this field in order to comprehensively describe such criteria.

**Table 1:** Synthesis of optimal and suitable water flow velocity and depth values for macroinvertebrates based on literature.

Analyzed group of organisms	Analyzed habitats within the riverbed	Observed flow velocity values	Observed depth values	References
*Baetis sp.*	Riffles	0.30–0.70 m/s (range of suitability)	0.30 m (upper limit of suitability)	^46^
Naididae*,* *Nesameletus sp.,* Chironomidae, *Potamopyrgus antipodarium*	All habitats	< 0.60 m/s (the highest density)	< 0.75 m (the highest density)	^40^
Trichoptera	–	< 0.60 m/s (upper limit of suitability)	–	^81^
The most frequent taxa	All habitats	< 0.60 m/s (the highest density)	0.20–0.30 m (the highest density)	^73^
All macroinvertebrates	All habitats	0.05–0.40 m/s (the highest density)	0.05–0.20 m (the highest density)	^34^
All macroinvertebrates	Riffles	0.65 m/s (the highest density)	0.27 m (the highest density)	^82^
All macroinvertebrates	All habitats	0.10–0.60 m/s (range of suitability)	0.10–1.00 m (range of suitability)	^24^
All macroinvertebrates	All habitats	< 0.75 m/s (the highest density)	< 0.60 m (the highest density)	^48^

Habitat suitability criteria for FFGs were similar to HSCs for the macroinvertebrate community, particularly for grazers and gatherers. This indicated that the shape of the curves was strongly influenced by the most abundant taxa. Nevertheless, the suitability values for the analyzed environmental parameters can be related to the mechanism of food intake in each group and their environmental availability. For example, grazers gnaw and scrape periphyton from different surfaces. The lower values of water flow velocity and depth may affect the presence of macrophytes that provide substrate for the epiphyton^28^. Small depths may also be suitable for grazers because light reaches primary producers more easily. In zones with higher velocities (e.g., within riffles), food may be located on larger debris grains (epilithon)^47^. Filter feeders prefer higher water velocities, which provide suspended organic matter (for this reason, substrate type is less important). The HSCs for predator were similar to the criteria for filter feeders and shredders. It should be noted, however, that many of the identified taxa are opportunistic organisms that use several mechanisms for food intake^83^. Therefore, some criteria may simultaneously consider parameters’ suitability for organisms belonging to several guilds. For example, gatherers are adapted to consume fine particle organic matter that is deposited on the surface of the sediment or in its deeper layers at sites of lower water flow velocities. These sites typically have shallow to deep depths with fine substrate (silt, fine sand). However, HSCs also showed the suitability of higher values of water flow velocity and coarse material, which may be due to the fact that some gatherers simultaneously obtain food by gnawing (e.g., *Baetis* sp., *Ephemerella* sp.)^63^. As another example, some shredders obtain food by gnawing in addition to shredding coarse particle organic matter (e.g., *Lymnaea* sp.)^63^. Nevertheless, the interpretation of the HSCs for FFGs would confirm that animals may indirectly respond to hydraulic conditions through food availability. Thus, relying solely on the relationship between organism distribution and hydraulic parameters may not reflect the actual preferences of organisms for specific habitats^43,44^. Consequently, when organic matter cycling in the riverbed is disturbed, the HSCs determined without consideration of ecological processes may be violated by disturbances in the distribution of organisms.

It was also observed that the shape of the HUCs and HPCs for filter feeders and the curves for the 2019 data for grazers, shredders, and predators were not as wide as for the rest of the analyzed groups, including the macroinvertebrate community. In the case of filter feeders, this is due to a preference towards high water flow velocities, which is seen in curves based on data from both 2018 and 2019. In 2019 the highest flow velocity values were smaller in comparison to 2018 due to lower flow. However, for grazers and shredders, the curves indicate clustering, which occurs during low water flows^35^. It may be that for shredders, lower water flow velocity values provide an opportunity for organisms to exist and find food closer to the central part of the channel where medium sand predominates (e.g., velocity values are sufficient for coarse particulate organic matter deposition). Based on the curves for 2019, shredders were assigned the widest range of substrate suitability. This would imply that for some groups (shredders, filter feeders, and predators), substrate suitability is a parameter that varies over time depending on prevailing flow conditions and food availability^84^. This statement again would undermine the aforementioned independence of the environmental parameters analyzed ^78^.

The analyses conducted confirmed the necessity and practical utility of performing detailed analyses of HSCs for FFGs and considering the curves determined separately for two field campaigns. Both facilitated the interpretation of processes that may have influenced the distribution of organisms, especially due to changes of flow conditions. Thus, it is highly advisable to consider both elements in future environmental flow assessments. First, including FFGs will make it possible to avoid problems associated with the influence of the most abundant taxa on HSC values. Additionally, FFGs will contribute to identification of the most vulnerable group in terms of flow rate. In our study, these were the filter feeders (they had the narrowest range of suitability towards flow velocities). Second, analyzing the data from several field campaigns separately will allow for estimation of more robust HSCs. Combining data without this step may result in erroneous determination of the range of suitability for the environmental parameters analyzed. Thus, the insights presented here are important for the protection of aquatic ecosystems and water management, as choosing the target group of organisms appropriately and determination of proper HSCs provide more robust environmental flow assessments. Importantly, analyses conducted in our study are suitable for different types of rivers, and information about FFGs is publicly available. This may contribute to a wider use of the approach outlined here. Nonetheless, despite the importance of the results obtained, we also recognize the need for further research. Due to the fact that macroinvertebrates are opportunistic organisms that use several mechanisms for food intake, it would be worth verifying other ecological traits. An alternative could be, for instance, to analyze the occurrence within the substrate: organisms that continuously inhabit the surface of the substrate (obligates), organisms that live most of the time on the surface of the substrate but also have the ability to move into the substrate (facultatives), and macroinvertebrates that avoid contact with open water (avoiders)^47^. Our results for HPCs and HUCs determined separately for data collected in 2018 and 2019 also showed that suitability regarding substrate type varied between seasons; thus we hypothesize that occurrence within the substrate could be a good choice.

Finally, our results may be important in the face of climate change. Given the changing hydro-climatic conditions, reliance on averaged ecological regimes and responses will, in many cases, be inadequate for estimating relationships between environmental parameters and the distribution of aquatic organisms^4^. This may be valid, even though it is likely that rivers might spontaneously adapt to prevailing climatic-hydrological conditions in the future, or even evolve to completely new, resilient aquatic ecosystems^85^. The mechanisms by which climate change affects organisms will depend on many factors, including species characteristics and regional conditions^86^. Nevertheless, common macroinvertebrates have already shown a markedly negative response to reduced flow in contrast to rare and moderately frequent species^87^. In addition to a shift in macroinvertebrate community structure toward eurytopic species^88^, climate change may affect the structure and density of FFGs^86^, and a greater influx of invasive species, which show greater tolerance to higher temperatures and lower oxygen content^85,86,89^. For instance, Jourdan et al. (2018) reported that grazers and scrapers were found to be the group most vulnerable to higher temperatures and reductions in stream flow. Also a negative impact of increasing temperatures and reduced precipitation on shredders was observed^86^. Consequently, disruption of FFG structure will lead to disruption of organic matter cycling in riverbeds and food webs. In contrast to Jourdan et al. (2018), in our study, the filter feeders were the group most demanding with respect to flow rate. Their abundance and variety significantly changed between the studied seasons and they had the narrowest suitability towards flow velocities. The differences detected may indicate variability in response to changing hydro-climatic conditions. Therefore, we hypothesize that a detailed interpretation of HSCs with a background of ecological processes will be crucial to address the challenges of a changing ecosystem structure^4^.

## Conclusions

A detailed analysis of habitat suitability curves for a macroinvertebrate community and FFGs led us to several insights and conclusions. We summarize our key findings in the following points:

- A key element in the determination of the HSCs was the analysis of the HPCs and HUCs separately for the data collected in 2018 and 2019. The results showed the importance of acquiring data at two different flows, which had not been considered relevant for determining habitat suitability criteria. Usually it is advised to conduct a single field campaign to avoid the problems of combining data. However, multiple field campaigns make it possible to capture a wider range of environmental conditions and organism distributions, which may not be noted at a single flow rate. In addition, separate curves for each campaign may show the suitability of environmental parameters under different conditions, and ranges of this suitability may differ from the values obtained for the combined data.
- Analyzing HSCs for guilds of macroinvertebrates so far has not been encountered in the literature. This step facilitated the understanding of mechanisms that influenced the distribution of organisms. It also showed that the shape of HSCs for the macroinvertebrate community may be influenced by the most abundant taxa (in this case grazers and gatherers). This means that information on the suitability of environmental parameters for less abundant, but more vulnerable taxa in terms of environmental parameters may be averaged out or overlooked. Nevertheless, due to fact that macroinvertebrates are opportunistic organisms in terms of food intake, further studies should be carried out to include other ecological traits.
- The comparison of the resultant HSCs and literature data suggests that it is reasonable to determine the universal habitat suitability criteria, since both sources showed a similar range of optimal and suitable values of the analyzed environmental parameters. However, variability in response to changing hydro-climatic conditions may mean that future HSCs will have to be more specific in terms of vulnerable groups of organisms.
- Analysis of the K parameter and values of resultant curves showed that HUCs coincided with the upper limit of the K parameter but for HPCs the discrepancies were larger, which may be caused by the fact that the K parameter does not take into account information about the availability of habitats. Results obtained for FFGs suggested that the biodiversity indices used in calculation of the K parameter may not be the best measure for estimating habitat suitability for smaller ecological groups.
- Due to the influence of substrate stability on the distribution of some of the FFGs, future analyses should consider the implementation in HSCs of parameters related to sediment transport processes and substrate stability.
- Distribution of macroinvertebrates and convergence with environmental parameters of specific habitat types suggest that the assumption of independency in univariate curves is not valid.

## Supplementary Information

Supplementary Information.

## Data Availability

The datasets used and/or analyzed during the current study are available from the corresponding author on reasonable request.
